# Mapping the Evidence on Cardiovascular Disease Risk Among Individuals Experiencing Disability: A Scoping Review

**DOI:** 10.1097/PHM.0000000000003018

**Published:** 2026-05-06

**Authors:** Yan Xu, Elena Koelbener, Oscar H. Franco, Beatrice Minder, Inge Eriks-Hoogland, Adrian Martinez-De la Torre, Carla Sabariego, Marija Glisic

**Affiliations:** 1 Swiss Paraplegic Research, Nottwil, Switzerland; 2 Institute of Social and Preventive Medicine (ISPM), University of Bern, Bern, Switzerland; 3 Utrecht University, Julius Center for Health Sciences and Primary Care, University Medical Center Utrecht, Utrecht, Netherlands; 4 Public Health and Primary Care Library, University Library of Bern, University of Bern, Bern, Switzerland; 5 Swiss Paraplegic Center, Nottwil, Switzerland; 6 Faculty of Health Sciences and Medicine, University Lucerne, Lucerne, Switzerland

**Keywords:** Cardiovascular Disease, Disability, Risk Factors, Stroke

## Abstract

**Objective:**

To map the existing evidence on CVD risk among individuals with complex and disabling health conditions, including traumatic brain injury (TBI), multiple sclerosis (MS), spinal cord injury (SCI), cerebral palsy (CP), spina bifida (SB), and poliomyelitis, and identify gaps to guide future research.

**Design:**

A scoping review was conducted following an established framework and preregistered protocol. We searched MEDLINE, Embase, Web of Science, and Google Scholar from inception until March 17, 2025. Data on study characteristics, populations, outcomes, and risk factors were extracted and synthesized descriptively.

**Results:**

A total of 38 observational studies published between 2001 and 2024, comprising more than 1.6 million individuals, met the inclusion criteria. Most studies compared these populations with the general population, focusing on stroke-related outcomes, and revealed a limited, fragmented, and geographically uneven evidence base with little attention to within-group determinants.

**Conclusions:**

Overall, the literature reflects growing interest in cardiovascular risk among individuals with complex and disabling health conditions but is limited by methodological heterogeneity, uneven condition representation, and reliance on administrative data. Future research would benefit from standardized disability definitions using consistent diagnostic criteria, improved reporting of patient-level factors, and broader representation across impairment groups and regions.

**What Is Known**

- Individuals with complex disabling conditions face elevated cardiovascular risk, but existing evidence is scattered and incomplete.
**What Is New**
- This manuscript provides the first scoping review to systematically map the scale and characteristics of evidence on CVD risk across complex and disabling health conditions. Synthesizing data from 38 observational studies including over 1.6 million individuals, we demonstrate that the current evidence base is uneven, with certain disability etiologies disproportionately represented and substantial variability in the outcomes assessed across studies. Importantly, we identify major gaps in understanding modifiable risk factors and within-group determinants directly relevant to rehabilitation practice and long-term health management.

Disability linked to conditions such as traumatic brain injury (TBI), multiple sclerosis (MS), spinal cord injury (SCI), cerebral palsy (CP), spina bifida (SB), and poliomyelitis (polio) affects millions of people worldwide.^1–4^ As survival rates and life expectancy continue to improve,^5^ number of populations is growing and increasingly aging with complex and disabling health conditions.^6^

Aging with complex and disabling health conditions presents unique health challenges: beyond the primary neurological impairment, individuals often experience reduced mobility, chronic low-grade inflammation, and increased oxidative stress, which are major factors associated with increased cardiovascular risk.^7^

Cardiovascular disease (CVD) is therefore of particular concern in these groups. For instance, individuals with SCI experience markedly increased cardiometabolic risk within 6 months postinjury.^8,9^ Longitudinal evidence also shows a rapid accumulation of secondary health conditions during the transition from rehabilitation to community living,^10^ reporting a 5-year prevalence and incidence of any cardiometabolic disorder exceeding 50%.^11^ Similarly, individuals with MS have higher risk of acute coronary syndrome, cerebrovascular disease, and cardiovascular mortality compared with matched controls.^12^ Individuals with TBI also exhibit substantially increased long-term risk of coronary and cerebrovascular events, as demonstrated in large longitudinal and population-based studies.^13,14^

Additional disparities linked to age, sex, and geographical location further shape CVD susceptibility and outcomes, suggesting that cardiovascular risk in individuals with complex and disabling health conditions is multifactorial and not yet fully understood.^15,16^ Despite growing awareness, the overall scope, depth, and methodological quality of existing evidence on the association between physical impairment and CVD remain unclear.

The primary objective of the current study was to map the scope and characteristics of existing research on the association between disability and CVD risk.

To achieve this objective, the review addressed the following specific questions:

1. What is the scope and nature of existing research on the association between disability and CVD?
2. Which populations with disability have been studied in relation to CVD risk?
3. What types of CVD outcomes are commonly investigated in relation to disability?
4. What methods and study designs are used to assess this association?
5. And what are the key methodological concerns identified in the existing literature?

By mapping the current evidence, identifying what methodological approaches are used, and where key limitations remain, this review will clarify the current state of evidence and highlight priority areas for future research.

## METHODS

This study followed the scoping review framework by Arksey and O’Malley,^17^ a structured process involving the following steps: (1) research question formulation, (2) identification of relevant articles, (3) study selection, (4) data extraction, and (5) data analysis and presentation of the findings. The review has been reported in accordance with the PRISMA-Scoping Reviews guideline. The PRISMA checklist is presented within the online appendix, Supplemental Digital Content 1, http://links.lww.com/PHM/C965 ^18^. The scoping review protocol has been registered in Open Science Framework https://osf.io/2evjs/overview.

### Search Strategy and Sources

A comprehensive literature search was conducted in collaboration with an experienced librarian across MEDLINE (Ovid), Embase (Elsevier), and Web of Science Core Collection (Clarivate) from database inception to March 17, 2025, using a predefined search strategy (see Appendix 1, Supplemental Digital Content 1, http://links.lww.com/PHM/C965). No restrictions were applied regarding publication date or language. To validate the comprehensiveness of the strategy, the first 200 results from Google Scholar were also reviewed. Duplicate records were removed using Deduklick,^19^ a fully automated deduplication solution.

The search strategy included terms related to physical disability and CVD, including “physical disability,” “disabled person,” “paraplegia,” “heart disease,” “cardiovascular disease,” “risk,” “prevalence,” and “incidence,” as detailed in the Appendix, Supplemental Digital Content 1, http://links.lww.com/PHM/C965.

Search results were imported into Covidence for title and abstract screening, followed by full-text review. In addition, reference lists of all included studies were manually screened to identify any additional eligible publications.

### Study Criteria

The study selection criteria were developed using the PECOS (Population, Exposure, Comparator, Outcome, and Study) framework. Disability encompasses physical, mental, or functional impairments that restrict an individual’s capacity to perform daily activities and participate fully in life. In this review, we focus specifically on physical and functional impairments,^20^ or namely the following health conditions: TBI, MS, SCI, CP, SB, and Polio.

Longitudinal studies comparing individuals with and without disabilities or exploring CVD risk factors within individuals with physical disabilities were eligible. The aim was to identify observational studies examining the incidence of CVD, with a focus on 3 primary outcomes: stroke, myocardial infarction (MI), and heart failure (HF). Studies were excluded if they used CVD risk prediction tools (eg, Framingham Risk Score, QRISK3), or if they were interventional, cross-sectional, animal, or in vitro studies. Also excluded were reviews, case reports, case series, commentaries, letters, and conference abstracts.

### Study Selection and Data Extraction

Two reviewers (YX and EK) independently screened titles and abstracts, as well as the followed full-text screening of potentially eligible articles. Disagreements were resolved through discussion, or, if needed, reviewed by a third reviewer. Each reviewer examined all the articles separately and then documented the findings individually. Data extraction was performed using a predefined standardized form. Any discrepancies were resolved through discussion or consultation with a third reviewer. For each included study, information was extracted on general characteristics (first author, publication year, country or data source, study design, and setting), journal information (journal name, open access status, and total number of authors), study characteristics (population size, proportion of females, age distribution, and type of complex disabling health condition), outcome information (definitions and assessment methods for cardiovascular outcomes), analytical approaches (statistical methods applied), and additional details such as other reported outcomes and funding sources.

#### Data Summary and Synthesis of Findings

In this scoping review, we mapped and described the current research landscape examining the association between disability and CVD risk. The analysis summarized the different populations that have been studied in relation to CVD risk, along with the cardiovascular outcomes most frequently reported, such as stroke, myocardial infarction, and heart failure. Furthermore, we reviewed the study designs and analytical methods used to explore these associations, paying particular attention to the variability in methodological approaches. The review has also highlighted the major methodological considerations, including how physical impairment is defined, how outcomes are objectively measured, the length of follow-up, demographic characteristics (sex and age), and funding sources. Together, these elements have provided a comprehensive overview of the existing evidence base.

#### Patient and Public Involvement

Patients and members of the public were not involved in any aspect of the study’s design, implementation, analysis, or dissemination.

## RESULT

### Study Selection

**FIGURE 1:**
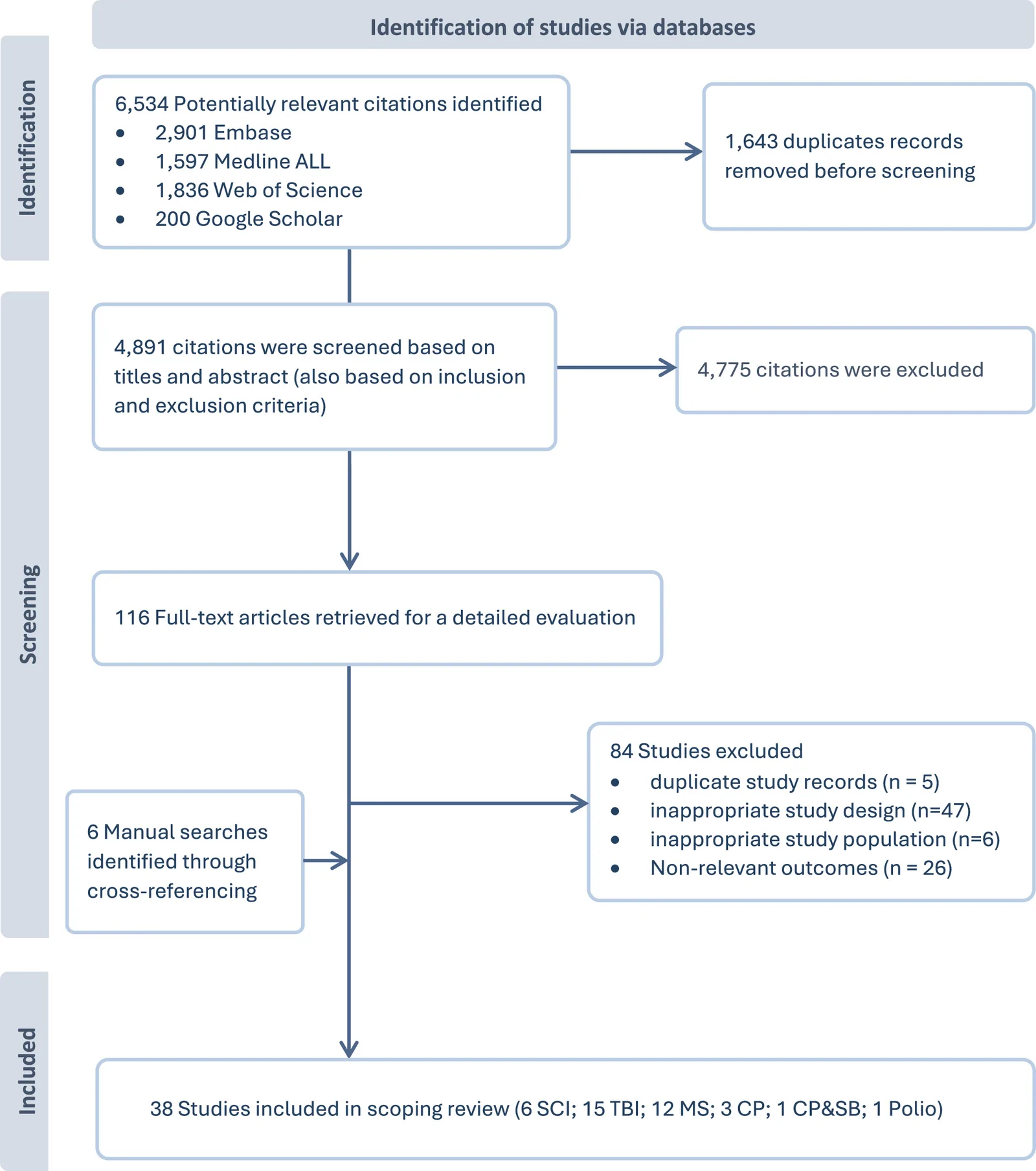
Flow chart of included studies. This figure shows the study selection process. Of 6534 records identified, 1643 duplicates were removed. After screening titles and abstracts, 4775 were excluded, followed by 84 after full-text review. In addition, we identified 6 citations through cross-referencing. In total, 38 studies were included in the scoping review, with 6 studies in SCI, 15 studies in TBI, 12 studies in MS, 3 studies in CP, 1 study in CP and SB, and 1 in polio.

**FIGURE 2:**
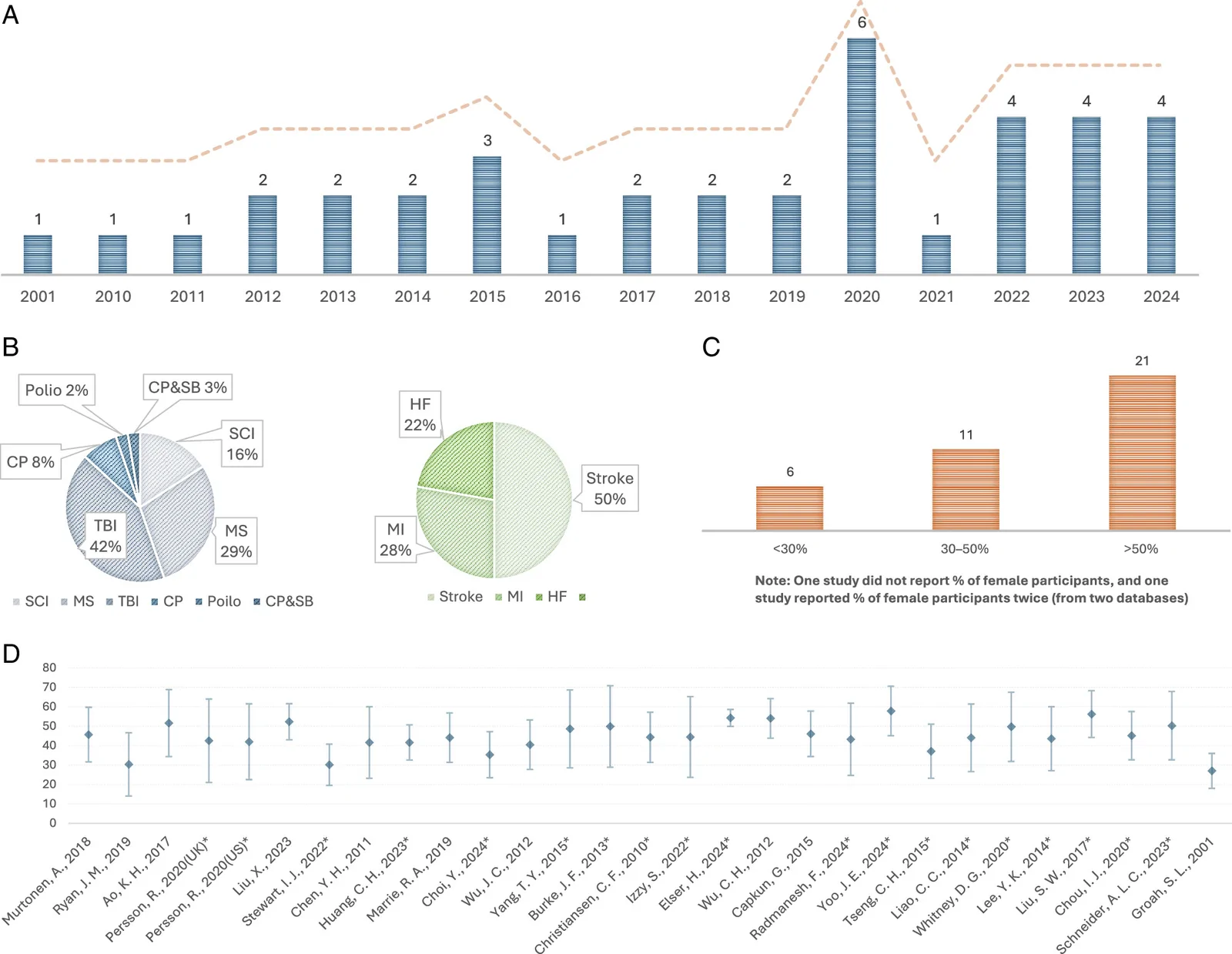
Summary of characteristics of included studies. This figure provides an overview of publication years (A), population groups (B), and sex (C) and age (D) characteristics of the 38 included studies.

A total of 6534 citations were identified through 4 databases. After removing duplicates, 4891 records were screened, and 116 full-text articles were assessed for eligibility, with 6 additional studies identified through cross-referencing. Eighty-four studies were excluded after review of the full text due to inappropriate study design (n=47) or study population (n=6) or being duplicate study records (n=5) or nonrelevant outcomes (n=26). Ultimately, 38 studies met the inclusion criteria and were included in this scoping review (Fig. 1). Studies were published between 2001 and 2024, and Figure 2A summarizes temporal publication trends.

### Population Focus and Representativeness

Table 1 summarizes the characteristics of the included studies. Most studies (n=16, 42.1%) focused on individuals with TBI, 11 (28.9%) on MS, 6 (15.8%) on SCI, 3 (7.9%) on CP, one (2.6%) on CP and SB, and one (2.6%) on Polio (Fig. 2B). Overall, the studies included 1,652,322 participants with complex and disabling conditions and 12,459,403 participants from the general population. Sex distribution varied, half of the studies included >50% women, 13 (34.2%) had 30% to 50% women, and 3 (7.9%) had <30%, typically in SCI populations; one study (2.6%) did not report sex distribution (Fig. 2C). Age reporting was inconsistent, either as mean ± SD or median (IQR). Nine studies (23.7%) included participants aged 18 to 44 years, 16 (42.1%) focused on middle-aged adults (45 to 64 y), one (2.6%) on older adults (≥65 y), and 11 (28.9%) reported age only in categories or incompletely (Fig. 2D).

**TABLE 1:** Extraction table summarizing the characteristics of included studies in this scoping review

Author	Year of Publication	Country	Study Design	Study Setting	Scientific Journal	Open Access Article	Total Number of Authors	Population Size	Percentage of Female Study Population	Population Age	Physical Disability	Outcome	Outcome Assessment	Statistical Analysis	Other outcomes	Funding
**Comparison to general population**																
Castelo-Branco et al.^21^	2020	Sweden	RMCS	ND	Multiple Sclerosis Journal – Experimental, Translational and Clinical	Y	10	4539 MS/47,527 GP	NA	NA	MS	MI/stroke/HF	MR	1. X^2^ test or Fisher exact test 2. Incidence/rate analysis (IR, IRR; regression model not specified)	1. Cardiovascular comorbidity (except stroke/MI/HF) 2. Noncardiovascular comorbidity	Celgene (study conducted and financed via IQVIA)
Murtonen, A et al.^22^	2018	Finland	RNCCS	HB	Multiple Sclerosis and Related Disorders	N	5	1074 MS/10740 GP	70.7%	45.67 (14.07)	MS	MI/stroke	MR	1. Kaplan-Meier and the log-rank test 2. X^2^ test	Other cerebrovascular diseas/sequelae of cerebrovascular disease/concomitant circulatory system diseases	The Finnish MS Society
Jadidi, E. et al.^23^	2013	Sweden	RMCS	ND (3)	Multiple Sclerosis Journal	N	3	7664 MS/ 66,215 GP	68.1% MS/ 68.9% GP	NA	MS	MI/stroke/HF	MR	Poisson regression	AF/Flutter	NO
Ryan, J. M et al.^24^	2019	UK	RMCS	ND	Neurology	Y	10	1705 CP/ 5115 GP	46.8%	30.33 (16.31)	CP	HF	MR	Cox proportional regression	Cancer/respiratory disease/diabetes/other CVDs/other Noncommunicable disease	Brunel University London.
Ao, K. H et al.^25^	2017	Taiwan	RMCS	ND	Sleep Medicine	N	6	1174 TBI /5870 GP	50.43%	51.61 (17.26)	TBI	Stroke	MR	1. *t* test or Mann-Whitney U-test 2. X^2^ test 3. Poisson regress 4. Cox proportional regression 5. Kaplan-Meier and the log-rank test	NA	NA
Nyam. T.T.E	2019	Taiwan	RMCS	ND	World Neurosurgery	Y	8	16211 TBI/32,422 GP	39.37% TBI/39.31% GP	NA	TBI	Stroke	MR	1. Kaplan-Meier and the log-rank test 2. Cox proportional regression	1. Death 2. CVDs	ChiMei Foundation Hospital Research (CMFHR10682) grants
Persson, R. et al.^26^	2020	UK & US	RMCS	ND	Multiple Sclerosis and Related Disorders	Y	9	5726 MS / 57,331 GP	71.9%	42.5 (21.5)^a^	MS	MI/stroke/HF	MR	1. Poisson regression (Byar’s method) 2. Kaplan-Meier and the log-rank test 3. X^2^ test or Fisher exact test	Other CV-events	Celgene
		UK & US		MHS				6406 MS/66,281 GP	71%	42 (19.5)^a^						
Liu, X et al.^27^	2023	China	RNCS	HB(11)	Reviews in Cardiovascular Medicine	Y	11	624 head injured/ 2496 GP	4.5%	52.3 (9.3)	TBI	MI/stroke	MR+self-reported questionnaires	1. Variance or the Kruskal- Wallis test 2. X^2^ test 3. Cox proportional hazard regression	All other sites injuries (head, chest, abdominal, extremities, others)	National Key R&D Program of China
Stewart, I. J et al.^28^	2022	USA	RCS	MHS (5) ND	JAMA Neurology	Y	13	301,169 TBI /1,258,759 GP	11.9% TBI/ 19.6% GP	30.15 (10.61)^a^	TBI	Stroke	MR	Fine-Gray competing risks models	Cardia death/PAD/CAD	1. The US Department of Defense 2. US Department of Veterans Affairs 3. Uniformed Services University.
Chen, Y. H et al.^29^	2011	Taiwan	RCCS	ND	Stroke	Y	3	23,199 TBI/ 69,597 GP	46.4%	41.6 (18.4)	TBI	Stroke	MR	1. Kaplan-Meier and the log-rank test 2. Cox proportional hazard regression	Hospitalization or health care utilization	NA
Huang, C. H.^30^	2023	Taiwan	RMCS	ND	PLoS One	Y	3	29,570 TBI/ 118,280 GP	44.3% TBI/ 43.75% GP	41.63 (9.07)^a^	TBI	HF	MR	1. Cox proportional hazard regression 2. Fine-Gray model	CHD	NO
Peterson, M. D.^11^	2021	US	RCS	PICD	The Spine Journal	Y	7	9081 SCI/ 147,4232 GP	57.8% SCI/ 52.5% GP	NA	SCI	HF	MR	1. Kaplan-Meier 2. Survival models 3. Weibull regression	Cardiometabolic morbidestes (except HF)	1. The National Institute on Disability, Independent Living, and Rehabilitation Research 2. the Craig H. Neilsen Foundation
Wu C. W et al.	2017	Taiwan	RMCS	ND	Developmental Medicine & Child Neurology	Y	6	1975 CP/9875 GP	53.9%	NA	CP	Stroke	MR	1. X^2^ test or Fisher exact test 2. Cox proportional hazard regression 3. Kaplan-Meier method	NA	NA
Marrie, R. A.^32^	2019	Canada	RMCS	ND (2)	Neurology Journals	N	8	14,565 MS /72,825 GP	73.1%	44.1 (12.7)	MS	MI	MR	Cox proportional hazard regression	NA	1. Waugh Family Foundation MS Society of Canada Operating Grant 2. the Waugh Family Chair in Multiple Sclerosis 3. a Research Chair from Research Manitoba
Choi, Y.^33^	2024	Korea	RMCS	ND	Journal of the American Heart Association	Y	7	518,423 TBI/GP	47.92%	35.33 (11.86)^a^	TBI	Stroke	MR	1. X^2^ test 2. Time-dependent Cox proportional hazard regression	NA	Ministry of Land, Infrastructure and Transport Research Fund
Cho, E. B.	2022a	Korea	RMCS	ND	Multiple Sclerosis Journal	N	7	1503 MS/ 7515 GP	60.1%	NA	MS	MI	MR	1. X^2^ test or Fisher exact test 2. Cox proportional hazard regression 3. Kaplan-Meier method	NA	Korea Health Tech R&D Project, KHIDI, Ministry of Health & Welfare, Korea (HI20C1073)
Wu, J. C^52^	2012	Taiwan	RMCS	ND	Neurology Journals	N	7	2806 SCI/28,060 GP	23.6%	40.48 (12.74)	SCI	Stroke	MR	1. X^2^ test and independent *t* tests 2. Kaplan-Meier 3. Log-rank test 4. Cox proportional hazard regression	Hemorrhagic stroke	NA
Yang, T. Y.^35^	2015	Taiwan	RMCS	ND	Spine Journal	N	4	22,197 SCI/ 88,788 GP	38.94%	48.63 (20.09)^a^	SCI	MI	MR	1. X^2^ test 2. Kaplan-Meier method 3. Log-rank test 4. Cox proportional hazard regression	NA	1. CMUH study projects & Cancer Research CoE 2. MOHW Clinical Trial & Research CoE 3. Health & Welfare Tobacco Surcharge
Burke, J. F.^51^	2013	USA	RCS	ND (4)	Neurology Journals	N	6	436,630 TBI/ 736,723 GP	46.8% TBI /49.3% GP	49.89 (20.99)^a^	TBI	Stroke	MR	1. Kaplan-Meier method 2. Log-rank test 3. Nelson-Aalen method 4. Cox proportional hazard regression	Stroke hospitalization	NA
Cho, E. B.	2022b	Korea	RMCS	ND	BMJ journal - Journal of Neurology, Neurosurgery and Psychiatry	N	7	1541 MS/ 7705 GP	60%	NA	MS	Stroke	MR	1. χ2 or Fisher exact test 2. Kaplan-Meier method 3. Log-rank test 4. Cox proportional hazard regression	Hemorrhagic stroke	KHIDI, Korea Health Tech R&D Project (HI20C1073)
Christiansen, C. F.^37^	2010	Denmark	RMCS	ND	Neuroepidemiology	N	6	13,963 MS/ 66,407 GP	64.1% MS/64.3% GP	44.3 (12.9)^a^	MS	MI/stroke/HF	MR	Cox proportional hazard regression	Atriral fibrillation/flutter	Merck Serono S.A. (Merck KGaA affiliate)
Izzy, S.	2022	USA	PMCS	HB	JAMA Netw Open	Y	20	8702 TBI/4351 GP	45%	44.45 (20.80)^a^	TBI	Stroke	MR	1. Cox proportional hazard regression 2. Kaplan-Meier 3. Log-rank test 4. Logistic regression	1. Chronic Cardiovascular, Endocrine, Neurological (except Stroke), and Psychiatric Disorders 2. Mortality	Novartis; Pfizer; Amgen; Bristol Myers Squibb; GE; Becton Dickenson; Boston Scientific; Mass General Brigham; Springer/Demos; Myomo; OnCare; Football Players Health Study at Harvard (NFLPA-funded)
Elser, H.^38^	2024	USA	PNCS	CB (4)	Stroke	Y	9	2158 TBI/10,655 GP	65.8% TBI/ 56.1% GP	54.28 (4.35)^a^	TBI	Stroke	MR + self-report (intervein)	1. Cox proportional hazard regression 2. Fine-Gray models	NA	NIH, NHLBI, NINDS, NIA, NIDCD
Wu, C. H.^39^	2012	Taiwan	PMCS	ND	Archives of Physical Medicine and Rehabilitation	Y	6	212 polio/424 GP	42.9%	54.0 (10.2)	Polio	Stroke	MR	1. χ2 or Fisher exact test 2. logistic regression	NA	1. Wan Fang Hospital 2. Center of Excellence for Clinical Trials and Research in Neuroscience
Capkun, G.^40^	2015	USA	RMCS	MHS	Multiple Sclerosis and Related Disorders	Y	14	15,684 MS /78,420 GP	76.5%	46.07 (11.7)	MS	Stroke/MI	MR	1. *t* test 2. Wilcoxon rank-sum test 3. χ2 or Fisher exact test 4. for Rate ratio analysis did not mention the analysis	Mortality and cause of death	Novartis Pharma AG,
Radmanesh, F.^41^	2024	USA	RCCS	HB	Neurotrauma Reports	Y	12	3664 TBI/ 1848 GP	50.3% TBI/ 47.5% GP	43.27 (18.61)^aa^	TBI	Stroke	MR	1. Logistic regression 2. Conditional Direct Effect, CDE	NA	Harvard Catalyst and the NFLPA
Yoo, J. E.^42^	2024	Korea	RMCS	ND	Journal of the American College of Cardiology	Y	8	5083 SCI/19,320 without SCI	25.2% SCI/ 24.8% GP	57.86 (12.75)^a^>	SCI	MI/HF	MR + self-administered questionnaires	1. χ2 test 2. Cox proportional hazard regression	Atrial fibrillation	KHIDI/Korea Health Technology R&D Project (HI20C1073)
Tseng, C. H.^43^	2015	Taiwan	RMCS	ND	European Journal of Neurology	N	4	1174 MS /4696 GP	78.1%	37.1 (13.91)^a^	MS	Stroke	MR	1. χ2 and *t* test 2. Cox proportional hazard regression 3. Kaplan-Meier 4. Log-rank test	NA	1. Taiwan Ministry of Health and Welfare Clinical Trial & Research Center of Excellence 2. China Medical University Hospital 3. Academia Sinica Taiwan Biobank 4. Stroke Biosignature Project 5. NRPB Stroke Clinical Trial Consortium 6. Tseng-Lien Lin Foundation, Taiwan 7. Taiwan Brain Disease Foundation 8. Katsuzo & Kiyo Aoshima Memorial Funds, Japan
Liao, C. C.^44^	2014	Taiwan	RMCS	ND	Mayo Clinic Proceedings	N	6	30,165 TBI /120,660 GP	49.6%	44.02 (17.40)^a^	TBI	Stroke	MR	1. χ2 test 2. Cox proportional hazard regression 3. Multivariate logistic regression	Poststroke mortality risk	1. Foundation for Anesthesia Education and Research 2. Taiwan Department of Health Clinical Trial & Research Center of Excellence
Whitney DG^45^	2020	USA	RCS	PICD	Bone	N	4	8345 CP/ 4758124 GP	47.3%CP/52.3% GP	49.70 (17.8)^a^	CP	HF	MR	Cox proportional hazard regression	Any CVD/ Ischemic heart disease/ Cerebrovascular disease	1. University of Michigan Office of Health Equity and Inclusion Diversity Fund 2. American Academy of Cerebral Palsy and Developmental Medicine
Peterson M.D^46^	2020	USA	RCS	PICD	The American Journal of Medicine	N	7	15,302 CP or SB/ 1,935,480 without CP or SB	56.6% with /52.3% without	NA	CP and SB	HF	MR	1. Kaplan-Meier 2. multiple Survival models 3. Weibull regression	Any cardiometabolic incidents (except HF)	National Institute on Disability, Independent Living, and Rehabilitation Research
Lee YK^50^	2014	Taiwan	RCS	ND	Scandinavian Journal of Trauma, Resuscitation and Emergency Medicine	Y	5	24,905 TBI/ 719,811 GP	52.6% TBI/51.5% GP	43.59 (16.44)^a^	TBI	Stroke	MR	1. χ2 and *t* test 2. Cox proportional hazard regression	NA	NA
Liu SW^47^	2017	Taiwan	RMCS	ND	International Journal of Environmental Research and Public Health	y	10	13,652 TBI/GP	54.1% TBI/54% GP	56.25 (12.06)^a^	TBI	Stroke	MR	1. χ2 and *t* test 2. Kaplan-Meier and log-rank test 3. Cox proportional hazard regression	Hemorrhagic stroke	NA
Chou IJ et al.^48^	2020	UK/ CPRD	RMCS	ND	European Journal of Neurology	Y	7	2526 MS/9980 without MS	70.86% MS/70.84% GP	45.14 (12.43)^a^	MS	HF/MI	MR	1. Logistic regression 2. Kaplan-Meier 3. Cox proportional hazard regression	Other Comorbidities (except HF/MI)	1. Chang Gung Memorial Hospital 2. University of Nottingham
Schneider. ALC^49^	2023	USA	RMCS	MHS (2)	Storke	Y	6	306,796 for both	9%	50.3 (17.6)^a^	TBI	Stroke	MR	1. Kaplan-Meier 2. Cox proportional hazard regression 3. Fine-gray proportional hazard regression	Hemorrhagic stroke	1. US Department of Defense (Psychological Health/Traumatic Brain Injury Research Program) 2. the US Department of Veterans Affairs, and the 3. National Institute on Aging.
**Within complex and disabling conditions**																
McFarlane, T. D.^53^	2020	USA	RCS	Registry	The Journal of Head Trauma Rehabilitation	N	5	58294 TBI	50.9%	NA	TBI	Stroke	MR	1. χ2 test 2. Cox proportional hazard regression	NA	The Indiana Spinal Cord & Brain Injury Research Fund from the Indiana State Department of Health
Meade, M. A.^54^	2023	USA	RCS	PICH	Mayo Clinic Proceedings	Y	7	8182 TSCI (2567 with T2D/5615 no T2D)	58.0% type 2 diabetes (T2D)/57.7% No T2D	NA	SCI	MI/stroke/HF	MR	1. χ2 test 2. Kaplan-Meier 3. Cox proportional hazard regression 4. Multivariable logistic regression	Long-term secondary Conditions (except Stroke/MI/HF)	1. National Institute on Disability, Independent Living, and Rehabilitation Research 2. The Craig H. Neilsen Foundation 3. The Fraternal Order of Eagles
Groah, S. L.^55^	2001	UK	RCS	HB (2)	Spinal Cord	Y	5	545	14%	27 (9)	SCI	MI	MR + interview	1. ANOVA 2. Kaplan-Meier and log-rank test	1. All CVD 2. CHD 3. Coronary atherosclerosis 4. Hypertension 5. Cerebrovascular disease 6. Dysrhythmia 7. Valvular disease	NA

^a^ Calculated from median values using a mean estimator.

This table presents key information extracted from each eligible study, including publication details (author, year, country), study design and setting, journal characteristics, authorship, and population characteristics (sample size, percentage of female participants, and age). The table also summarizes variables related to physical impairment, primary outcomes, outcome assessment methods, statistical analyses, other reported outcomes, and funding information.

Study setting / data source: CB=Community-based; HB=Hospital-based; MHS=Military Health System; ND=National Database-based Study; PICH=Private Insurance Claims Database; MR=Medical Record.

NA, not available; NR, not reported; PNCS, Prospective Nested Cohort Study; RCCS, Retrospective Case-Control Study; RCS, Retrospective Cohort Study; RMCS, Retrospective Matched Cohort Study; RNCCS, Retrospective Nested Case-Control Study; RNCS, Retrospective Nested Cohort Study; Study design: PCS, Prospective Cohort Study.

### Outcome Selection and Exposure Ascertainment

Most studies (n=34, 89.5%) assessed outcomes using medical records alone, while 4 (10.5%) combined records with self-reported questionnaires or interviews. The eligibility criteria and definitions for TBI, MS, SCI, CP, SB, and Polio are provided in the Supplementary Table, Supplemental Digital Content 1, http://links.lww.com/PHM/C965. Across the 11 MS studies, definitions varied, though most relied on diagnostic codes (ICD-8/9/10 or Read codes), with ICD-10 G35 being the most common. Some required repeated records or multiple claims, registry confirmation (Rare Intractable Disease program), or validation by McDonald criteria; one reported a positive predictive value of 94%. A few included related demyelinating diseases, and some required continuous enrollment before diagnosis. TBI definitions also differed widely, typically using ICD-9-CM codes (800–804 for skull fractures, 850 to 854 or 959.01 for intracranial injuries) and occasionally ICD-10 equivalents. Several applied CDC criteria to distinguish mild (850.x) from moderate-to-severe (851–854) TBI, while others used registry-based algorithms (DoDTR/TMDS, VA evaluations) or expert review. Some studies incorporated self-report, loss of consciousness, or injury cause via ICD-E codes. SCI was generally defined using ICD-9-CM codes 806.x or 952.x and ICD-10 cervical, thoracic, or lumbar codes, sometimes supplemented by registry confirmation, disability status, or neurological classification (Frankel/ASIA). CP was identified by ICD-9-CM 343.0 to 343.9 or Read codes; SB by ICD-9-CM 741.00 to 741.93; and poliomyelitis by ICD-9-CM 138. Overall, definitions ranged from simple administrative coding to clinically validated or registry-based criteria, contributing to heterogeneity across studies.

#### Study Design and Data Sources

**FIGURE 3:**
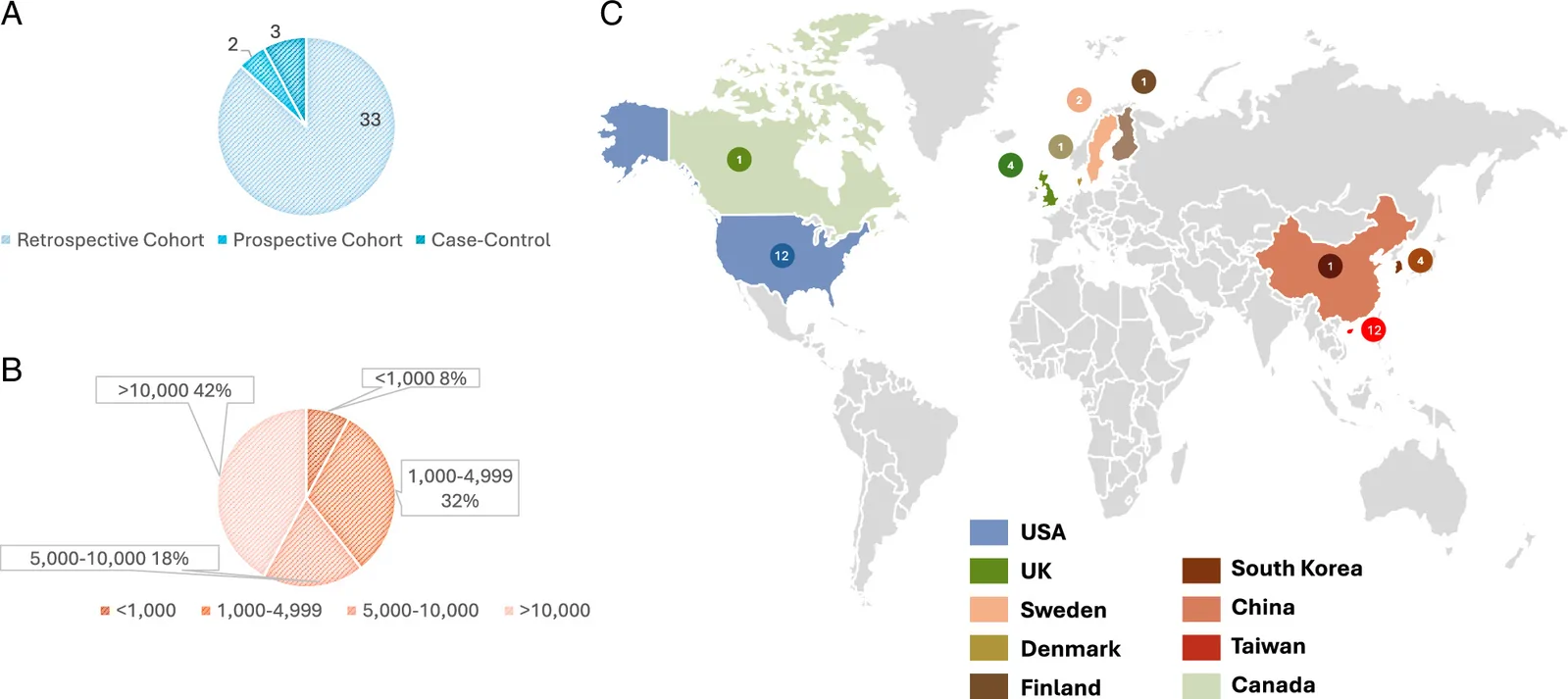
Summary of characteristics of included studies. This figure shows study design categories (A), sample size ranges (B), and geographical distribution (C) of the 38 included studies.

Most studies were designed as cohort studies (n=35, 92.1%), including 32 retrospective (84.2%) and 3 prospective cohorts (7.9%), while the remaining 3 (7.9%) were case-control studies (Fig. 3A). A majority (n=35, 92.1%) compared CVD risk with that in the general population, whereas 4 studies (10.5%) examined CVD risk factors within individuals with physical impairment. Sample sizes ranged widely: 3 studies (7.9%) enrolled <1000 participants; 12 (31.6%) had 1000 to 4999; 7 (18.4%) had 5000 to 10,000; and 16 (42.1%) included >10,000 participants, reflecting the use of administrative and registry-based health data (Fig. 3B). Most studies (n=33, 86.8%) used national databases, such as health insurance registries, inpatient/outpatient records, or population-wide registers from Taiwan, Korea, the US, Sweden, and the UK; a smaller number were hospital-based (n=3, 7.9%) or community-based (n=2, 5.3%).

The studies were conducted across multiple regions, with the highest numbers from the United States (n=12, 31.6%) and Taiwan (n=12, 31.6%), followed by the United Kingdom (n=4, 10.5%), South Korea (n=4, 10.5%), Sweden (n=2, 5.3%), Denmark (n=1, 2.6%), Finland (n=1, 2.6%), China (n=1, 2.6%), and Canada (n=1, 2.6%), indicating research concentration in North America and East Asia (Fig. 3C).

#### Evidence Sources and Funding

The studies appeared in a range of journals, with most focused on neurology, multiple sclerosis, cardiovascular medicine, general medicine, and rehabilitation or interdisciplinary fields. The top 5 journals were Stroke (n=3, 7.9%), Neurology (n=3, 7.9%), Multiple Sclerosis Journal (n=3, 7.9%), Multiple Sclerosis and Related Disorders (n=3, 7.9%), and Mayo Clinic Proceedings (n=2, 5.3%). Over half of the studies (n=20, 52.6%) were published open access. The number of authors per study ranged from 3 to 20 (median number of 7 authors), and most (n=24, 63.2%) had 5 to 10 authors.

Funding sources varied by country. US studies were mainly supported by federal agencies, private foundations, pharmaceutical companies, and academic institutions. Taiwanese studies received funding from hospitals, government agencies, national research institutions, and foundations, although several reported no or unclear funding. Korean studies relied on government R&D programs, U.K. studies on universities, and single studies in Finland, China, Canada, and Denmark received support from national societies, R&D programs, or industry, 2 studies (5.3%) reported no specific funding.

### Strategies to Address Association Between Physical Impairment and CVD Risk

The objectives of the included studies primarily focused on examining CVD risk-related outcomes in individuals with complex and disabling health conditions leading to physical disability, compared with the general population (n=35, 92.1%).^11,13,14,21–52^ A subset of studies (n=14, 36.9%)^21,25,26,31,35,38,40,42,43,45,49,53–55^ additionally aimed to identify demographic and clinical characteristics associated with increased CVD risk. These characteristics included age, sex, socioeconomic factors, comorbidities, and impairment-specific risk factors, such as injury level in SCI, disease severity in MS, and recurrent trauma in TBI.

Regarding outcomes, stroke was the most frequently investigated cardiovascular event (n=27, 71.1%^13,14,21–23,25–29,31,33,36–41,43,44,47,49–54^), followed by myocardial infarction (MI, n=14, 36.9%^21–23,26,27,32,34,35,37,40,42,48,54,55^) and heart failure (HF, n=12, 31.6%^11,21,23,24,26,30,37,42,45,46,48,54^). In addition to stroke, MI, and HF, the studies examined a broad range of other CVD-related outcomes (n=21, 55.3%), including atrial fibrillation/flutter, coronary heart disease, hypertension, other cerebrovascular and cardiometabolic events, noncardiovascular conditions (n=9, 23.7%) such as cancer, diabetes, chronic neurological or psychiatric disorders, as well as mortality (n=5, 13.2%), hospitalizations (n=2, 5.3%), health care utilization(n=1, 2.6%), and injuries (n=1, 2.6%). Overall, these outcomes reflect the diverse cardiovascular and noncardiovascular impacts considered in the studies.

Among the 35 studies comparing CVD risk with the general population that used matching, age and sex were the most consistently used factors. Other frequently matched variables included index date or year of diagnosis, region of residency, and race or ethnicity. Some studies additionally matched on comorbidities (eg, diabetes mellitus, hypertension, dyslipidemia), general practice, or propensity scores, while 6 studies did not report specific matching criteria. Overall, matching strategies focused primarily on demographic variables, with a minority incorporating clinical or health care-related factors.

A wide variety of statistical methods were applied across the studies. Survival analysis was most common, with Kaplan-Meier estimates and log-rank tests reported in over 20 studies, and Cox proportional hazards regression in more than 25 studies. Less frequently applied methods included Fine-Gray competing risks models, Weibull regression, and the Nelson-Aalen method. Categorical variables were typically analyzed using χ² or Fisher exact tests, while continuous variables were compared using *t* tests, Mann-Whitney *U* tests, ANOVA, or Kruskal-Wallis tests. Regression models were applied according to outcome type, including Cox regression for survival data, Poisson regression for incidence rates, and logistic regression for binary outcomes. Many studies combined approaches, with Kaplan-Meier and Cox regression forming the core of the analyses. Effect estimates were reported using HRs, IRRs, ORs, and RRs, with HRs being the most used.

## DISCUSSION

This scoping review identified 38 observational studies that examined CVD risk in individuals with complex and disabling health conditions, including TBI, MS, SCI, CP, SB, and polio. Most studies (92.1%) compared CVD risk in these populations with that of the general population, whereas relatively few examined within-group determinants or explored demographic, clinical, or mechanistic drivers of elevated risk.

Stroke was the most frequently assessed cardiovascular outcome, followed by myocardial infarction and heart failure. Many studies also reported a broad array of additional cardiovascular and noncardiovascular outcomes, such as atrial fibrillation/flutter, coronary heart disease, hypertension, other cerebrovascular or cardiometabolic events, cancer, diabetes, neurological or psychiatric disorders, mortality, hospitalizations, health care utilization, and injuries, reflecting the complex health challenges experienced by individuals with disabling health conditions.

Although more than 6500 citations were initially retrieved, fewer than 1% met the inclusion criteria, and directly addressed the research question. This underscores a sparse and fragmented evidence base on cardiovascular risk in individuals with lifelong or acquired physical impairments. Whereas the increasing number of publications over time suggests growing recognition of the cardiovascular burden in these groups, research efforts remain unevenly distributed across conditions and geographic regions, underscoring persistent gaps in knowledge.

### Critical Appraisal of Available Evidence

Most included studies used retrospective cohort designs, drawing primarily on national or administrative databases to capture large populations over extended follow-up periods. This approach supports robust estimation of population-level CVD risk but also introduces important limitations. Study populations were predominantly from the United States and East Asia, with very limited evidence from Europe and virtually none from other global regions. Such geographical concentration may restrict the generalizability of findings to populations with different sociodemographic characteristics, health care systems, and cultural contexts. Regional differences in disease epidemiology further reinforce this concern; for example, MS prevalence is substantially higher in North America and Northern Europe than in Asia and the Pacific.^2,56,57^ This heterogeneity underscores the need for research conducted in more diverse geographical and cultural settings to provide a comprehensive understanding of CVD risk in individuals with disabling conditions.

Sex and age distributions also varied widely across studies. Some cohorts were dominated by younger adults, particularly those with TBI, whereas others, such as MS, were more frequently middle-aged.^2,58^ Although women were well represented overall, sex distribution was strongly condition-specific, with male predominance particularly evident in SCI populations.^1^ Age was often reported inconsistently, either only in broad categories or incompletely, limiting meaningful comparison of age-related cardiovascular risk trajectories. Notably, certain conditions, such as CP, are predominantly studied in pediatric populations, which may contribute to the underrepresentation of adults with CP in the current literature. These demographic discrepancies highlight the heterogeneity of populations with physical impairments and complicate interpretation and synthesis of pooled evidence.

Marked heterogeneity was observed in the operational definitions of included health conditions. Most studies relied on administrative diagnostic codes, but the specificity and validation of coding strategies varied substantially across settings. Some required repeated claims, clinical validation, or registry confirmation, while others accepted single diagnostic entries without verification. Such variation may influence case ascertainment accuracy and introduce misclassification bias, particularly for conditions with evolving diagnostic criteria, such as MS, or with variable injury severity, such as TBI and SCI. Inconsistent reporting of disease severity or classification, such as SCI injury level or mild versus moderate-to-severe TBI, further contributes to heterogeneity and may obscure clinically meaningful gradients in cardiovascular risk. Although these differences reflect real-world data constraints, they highlight the need for standardized, validated diagnostic approaches in future research.

The reliance on administrative or registry datasets enabled large sample sizes, often exceeding 10,000 participants, and long-term follow-up. However, this design limits the availability of detailed clinical and behavioral information, including physical activity, diet, medication adherence, smoking, and physical functioning status. Only a small proportion of studies incorporated self-reported measures or clinical assessments, restricting the ability to explore mechanisms underlying elevated cardiovascular risk. Analytic strategies also varied, with survival methods, particularly Kaplan-Meier estimation and Cox proportional hazards regression, forming the core analytical approach. Matching procedures frequently accounted for age and sex but less often adjusted for comorbidities or health care utilization patterns, which may confound associations between physical impairment and CVD risk. Inadequate reporting of matching criteria in several studies further limits interpretability and comparability. Although effect measures were generally appropriate for the study designs, methodological variability complicates synthesis of risk estimates across conditions and settings.

Funding sources differed substantially by country. US studies were commonly supported by federal agencies or industry, while Taiwanese studies relied largely on hospital and governmental funding. Several studies reported no funding or unclear support, which may indicate limited prioritization of disability-related cardiovascular research in some regions. Such funding heterogeneity may influence research focus, sample availability, and methodological rigor.

### Suggestions for Future Research

Addressing current literature limitations will require longitudinal, multicenter studies that integrate administrative data with primary clinical, behavioral, and patient-reported measures such as cognitive impairment. Such approaches are essential to capture the full spectrum of cardiovascular risk factors, patient-level characteristics, identify high-risk subgroups, and understand patients lived experience, which will help to inform tailored prevention and management strategies. From a clinical and public health perspective, heightened awareness of CVD risk in individuals with complex neurological disabilities is needed, alongside research that supports more personalized, holistic models of care.^59^ Understanding how individual characteristics and impairment-specific factors influence outcomes may enable more targeted screening, monitoring, and intervention efforts, ultimately improving health and quality of life for affected individuals.

## CONCLUSIONS

Taken together, the evidence indicates substantial interest in understanding cardiovascular risk among individuals with complex and disabling health conditions, but the current literature is characterized by methodological heterogeneity, uneven condition and outcome representation, and heavy reliance on administrative data. These factors pose challenges for synthesizing findings and drawing firm conclusions about the magnitude and determinants of CVD risk across physical impairment populations. Nonetheless, consistent use of large datasets and robust analyses strengthens confidence in observed associations in more frequently studied conditions such as MS and TBI.

Standardization of diagnostic criteria, improved reporting of demographic and clinical characteristics, and incorporation of functional and behavioral risk factors are critical steps for advancing this field. Greater inclusion of underrepresented impairment groups and geographic regions will also be essential to developing equitable and generalizable cardiovascular prevention strategies.

## Supplementary Material
