# Supplementary material for: Mapping the Evidence on Cardiovascular Disease Risk Among Individuals Experiencing Disability: A Scoping Review
Source: Am J Phys Med Rehabil. 2026 May 6;105(10):938–50. doi: 10.1097/PHM.0000000000003018 (PMC13528847; doi:10.1097/PHM.0000000000003018)
Supplement: Supplementary file 1 [file phm-105-938-s001.docx]

**Supplementary Table. Definitions and diagnostic criteria of the conditions included in this review.**

Legend: This table summarizes the definitions, diagnostic criteria, and classification approaches used for each condition across the included 38 studies.

| **Condition** | **Studies** | **Definition and criteria** |
| --- | --- | --- |
| MS | Castelo-Branco 2020 | First primary MS diagnosis (ICD-10: G35), confirmed by a second primary MS diagnosis ≥181 days later |
|  | Murtonen A 2018 | MS diagnosis (ICD-10: G35), confirmed as definite MS by McDonald criteria (2011) |
|  | Jadidi E 2013 | First MS diagnosis (ICD-8: 340; ICD-9: 340; ICD-10: G35) |
|  | Persson R 2020 (UK/US) | First recorded MS diagnosis; ≥1 year enrollment prior to first MS diagnosis; cases validated via database-specific procedures; matched up to 10 non-MS patients by age, sex, date, and practice |
|  | Marrie R A 2019 | ≥3 claims for MS (ICD-9-CM 340 / ICD-10-CA G35) or related demyelinating diseases (optic neuritis, transverse myelitis, ADEM, NMO, etc.) |
|  | Cho E B 2022 (MI) | ≥2 outpatient or hospitalization claims with ICD-10 G35.0 plus registration in RID program (McDonald 2005 criteria) |
|  | Cho E B 2022 (Stroke) | ≥2 outpatient or hospitalization claims with ICD-10 G35.0 plus registration in RID program (McDonald 2005 criteria) |
|  | Christiansen C F 2010 | Hospital discharges since 1977; outpatient/ED visits since 1995; ICD-8 until 1993, ICD-10 thereafter; PPV = 94% |
|  | Capkun G 2015 | ICD-9-CM code 340.xx appearing ≥2 times, ≥30 days apart |
|  | Tseng C H 2015 | ICD-9-CM code 340; diagnosis from Jan 1, 1997 to Dec 31, 2010 |
|  | Chou I J 2020 | Read codes (validated; equivalent to ICD-10 340.0) |
| TBI | Ao K H 2017 | ICD-9-CM codes: skull fracture TBI (801–804) and non-skull fracture TBI (850–854); insomnia defined as ≥3 outpatient visits or 1 inpatient admission (ICD-9-CM: 307.4, 327, 780.5) |
|  | Nyam T T E 2018 | ICD-9-CM 800, 801, 803, 804, 850–853, 854.1, 959.01 |
|  | Liu X 2023 | Traumatic injury identified from hospital and insurance records; severity classified (mild, moderate, severe) by expert panel based on injury sites and vital signs |
|  | Stewart I J 2022 | Diagnosis prioritized via DoDTR/TMDS (GCS, AIS, ICD-9/10), VA comprehensive evaluation (LOC, amnesia), AFHSC algorithm |
|  | Chen Y H 2011 | ICD-9-CM codes 801–804 (skull fracture) or 850–854 (intracranial injury) |
|  | Huang C H 2023 | ICD-9-CM codes 800, 801, 803, 804, 850–853, 854.1, 959.01; |
|  | Choi Y 2024 | ICD-10 codes: S06.0–S06.9 (intracranial injuries), S02.0, S02.1, S02.7–S02.9 (skull fractures) |
|  | Burke J F 2013 | CDC TBI criteria: ICD-9-CM 800.0–801.9, 803.0–804.9, 850.0–854.1, 959.01 in any discharge field |
|  | Lzzy S 2022 | Mild TBI (mTBI) identified using CDC criteria; extracted via ICD-9 codes  Moderate-to-severe TBI (msTBI) identified using CDC criteria; extracted via ICD-9 codes  Cause classified using ICD-E codes: fall, motor vehicle, struck by/against, assault, other/unspecified |
|  | Elser, H.2024 | Self-reported head injury: year, number, loss of consciousness, care required. Hospitalized head injury: ICD-9 / ICD-10 codes |
|  | Radmanesh F 2024 | ICD-9: 800.0–801.9, 803.0–804.9, 850.0–854.1, 959.01, 807.0–807.9, 812–819.9, 822–822.9, or 823–827.9  ICD-10: S06.0X0A, S06.0X1A, S06.0X9A, S02.0, S02.10, S02.91, S06.1X, S06.2X, S06.30-S06.38, S06.4-S06.9 |
|  | Liao C C 2014 | ICD-9-CM codes: 800, 801, 803, 804, 850–854 Subtypes: skull fracture (800, 801, 803, 804), mild TBI (850), severe TBI (851–854); also assessed brain hemorrhage (852.x, 853) and loss of consciousness |
|  | Lee Y K 2014 | ICD-9-CM: 850.0, 850.1, 850.5, 850.9, 854.0, 959.01 Skull fractures (800.x, 801.x, 803.x, 804.x) |
|  | Liu S W 2017 | Concussion (first episode) ICD-9-CM: 850.x |
|  | Schneider A L C 2023 | ICD-9 and ICD-10 codes (Defense & Veterans Brain Injury Center list) |
|  | McFarlane, T. D.2020 | ICD-9-CM codes for TBI AIS ≥3 = serious TBI; AIS <3 = less serious TBI |
| SCI | Peterson M D 2021 | ICD-9-CM codes 806.00–806.9 (fracture with SCI) and 952.00–952.9 (SCI without spinal bone injury); hospitalization required |
|  | Wu J C 2012 | Hospitalization with ICD-9 806.X/952.X indicating permanent moderate-to-severe disability (paraplegia, tetraplegia, incontinence) and recorded in the catastrophic illness registry. |
|  | Yang T Y 2015 | ICD-9-CM 806 (vertebral fracture with SCI) or 952 (SCI without spinal fracture), with subgroups by spinal level: cervical (C), thoracic (T), and lumbar-sacral-coxal (L-S-Co). |
|  | Yoo J E 2024 | ICD-10 codes: cervical SCI (S14.0, S14.1), thoracic SCI (S24.0, S24.1), lumbar SCI (S34.0, S34.1, S34.4) |
|  | Groah, S. L. 2001 | Classified by neurological level (Frankel/ASIA) |
|  | Meade, M. A.2023 | ICD-9-CM/ICD-10-CM codes for TSCI |
| CP | Wu C W 2016 | ICD-9-CM codes 343, 333.7 |
|  | Ryan J M 2019 | Identified via 23 Read codes including spastic and congenital CP |
|  | Whitney D G 2020 | ICD-9/10 codes |
| CP&SB | Peterson M D 2020 | CP: ICD-9-CM codes 343-333.7  SB: ICD-9-CM codes 741.00-741.93 |
| Polio | Wu C H 2012 | ICD-9-CM code 138 |

**Appendix Search strategy terms**

**Embase.com** Concepts: (1) AND (2) AND (3) AND (4) - NOT (5) (Limits)

**1) Population: People with physical disabilities, including spinal cord injury**

('physical disability'/mj OR 'disabled person'/mj OR 'physically disabled person'/mj OR 'spinal cord injury'/exp/mj OR 'cervical spine injury'/mj OR 'spinal cord ischemia'/mj OR 'paraplegia'/mj OR 'spastic paraplegia'/mj OR 'quadriplegia'/mj OR 'spinal dysraphism'/mj OR 'spinal cord contusion'/mj OR 'spinal cord transsection'/mj OR 'spinal paralysis'/mj OR 'brain injury'/exp/mj OR 'paralysis'/exp/mj OR 'cerebral palsy'/mj OR 'muscular dystrophy'/exp/mj OR 'multiple sclerosis'/exp/mj OR 'poliomyelitis'/exp/mj OR ('injury'/exp/mj AND 'spinal cord'/exp/mj) OR 'para athlete'/exp/mj OR 'wheelchair athlete'/mj OR 'wheelchair user'/exp/mj OR (((physical*) NEAR/3 (disab* OR deficien* OR handicap* OR impair* OR incapac*)) OR ((spine or spinal) NEAR/3 (injur* or trauma* or damag* OR ruptur* OR paralys*)) OR ((brain OR cerebr*) NEAR/3 (injur* OR contus* OR concus* OR damag* OR lesion*)) OR ((individual OR individuals OR patient* OR person* OR people) NEAR/3 (paralys* OR disabilit* OR disabled)) OR ('spinal cord' NEAR/3 (lacerat* OR transect* OR lesion* OR trauma*)) OR (myelopath* NEAR/3 (trauma* OR post-trauma* OR posttrauma*)) OR parapleg* OR quadripleg* OR tetrapleg* OR 'spina bifida' OR paralympi* OR para-athlet* OR parathlet* OR para-sport* OR parasport* OR (athlet* NEAR/3 (disabilit* OR disabled)) OR ((wheelchair* OR wheel-chair*) NEAR/3 (user*)) OR (mobilit* NEAR/3 (disabilit*)) OR 'musculoskeletal disabilit*' OR ((muscle* OR muscular OR myoton*) NEAR/3 dystroph*) OR myodystroph* OR 'multiple sclerosis' OR 'cerebral palsy' OR 'cerebral paresis' OR poliomyelitis OR polio):ti,kw)

**2) Cardiovascular disease** (myocardial infarction, heart failure, stroke)

('cardiovascular disease'/mj OR 'heart disease'/mj OR 'vascular disease'/mj OR 'heart failure'/mj OR 'congestive heart failure'/mj OR 'heart infarction'/mj OR 'cerebrovascular accident'/mj OR (((cardiovascular OR cardiac OR heart) NEAR/3 (disease* OR disorder* OR event*)) OR cvd OR cvds OR ((heart OR cardia* OR myocard*) NEAR/3 (attack* OR fail* OR insufficien* OR infarct*)) OR stroke* OR cardiopath*):ti,kw) OR (('risk factor'/exp/mj OR 'risk'/mj OR risk*:ti,kw) AND ('cardiovascular disease'/exp/mj OR 'heart failure'/de OR 'heart infarction'/de OR 'cerebrovascular accident'/de))

**3) Risk (factors)**

('risk'/exp OR 'prevalence'/de OR 'incidence'/de OR (risk* or prevalence* or incidence* or predict* or associat*):ab,ti)

**4) Study type** (observational studies, cohorts, case-control studies, case-series)

('cohort analysis'/de OR 'longitudinal study'/de OR 'prospective study'/de OR 'retrospective study'/de OR 'follow up'/de OR 'case control study'/exp OR 'case study'/exp OR 'major clinical study'/de OR (cohort* OR ((prospectiv* OR retrospectiv* OR populat* OR observat* OR (case* NEAR/3 control*)) AND (stud*)) OR (case* NEAR/3 series) OR ((follow-up* OR followup* OR longitudinal*) AND stud*)):ab,ti)

**5) Limits** (excluding: conference abstracts, editorials, notes, case reports, (systematic) reviews, meta-analyses; pediatric studies, and animal studies)

**NOT** ([Conference Abstract]/lim OR [Note]/lim OR [Editorial]/lim)
**NOT** (('case report'/de OR case-report:ti) NOT ('cohort analysis'/exp OR 'case control study'/exp OR 'case study'/exp OR 'major clinical study'/de))

**NOT** ('systematic review'/de OR 'review'/de OR 'systematic review (topic)'/de OR 'meta analysis'/exp OR 'meta analysis (topic)'/exp OR ((systematic NEAR/3 review) OR meta-analys*):ti)

**NOT** ((child/exp OR pediatrics/exp OR (child* OR pediatr* OR infan*):ti) NOT (adult/exp OR (adult* OR elder*):ab,ti))
**NOT** ([animals]/lim NOT [humans]/lim)

**Medline ALL** (via Ovid)
<https://ovidsp.ovid.com/ovidweb.cgi?T=JS&NEWS=N&PAGE=main&SHAREDSEARCHID=sayvUbFe2gY3GUNe4yl4ywKPBBP7ERTP8LQEQ3SfZ68iAVnekz4AiqtpgQuOqSk8>

Concepts: (1) AND (2) AND (3) AND (4) - NOT (5) (Limits)

**1) Population: People with physical disabilities, including spinal cord injury**

(*Disabled Persons/ OR Amputees/ OR *Para-Athletes/ OR *Spinal Cord Injuries/ OR *Spinal Cord Ischemia/ OR *Paraplegia/ OR *Quadriplegia/ OR *Spinal Dysraphism/ OR exp *Brain Injuries/ OR *Paralysis/ OR *Cerebral Palsy/ OR *Muscular Dystrophies/ OR exp *Multiple Sclerosis/ OR *Poliomyelitis/ OR (*"Wounds and Injuries"/ AND *Spinal Cord/) OR (((physical*) ADJ3 (disab* OR deficien* OR handicap* OR impair* OR incapac*)) OR ((spine or spinal) ADJ3 (injur* or trauma* or damag* OR ruptur* OR paralys*)) OR ((brain OR cerebr*) ADJ3 (injur* OR contus* OR concus* OR damag* OR lesion*)) OR ((individual OR individuals OR patient* OR person* OR people) ADJ3 (paralys* OR disabilit* OR disabled)) OR (spinal cord ADJ3 (lacerat* OR transect* OR lesion* OR trauma*)) OR (myelopath* ADJ3 (trauma* OR post-trauma* OR posttrauma*)) OR parapleg* OR quadripleg* OR tetrapleg* OR spina bifida OR paralympi* OR para-athlet* OR parathlet* OR para-sport* OR parasport* OR (athlet* ADJ3 (disabilit* OR disabled)) OR ((wheelchair* OR wheel-chair*) ADJ3 (user*)) OR (mobilit* ADJ3 (disabilit*)) OR musculoskeletal disabilit* OR ((muscle* OR muscular OR myoton*) ADJ3 dystroph*) OR myodystroph* OR multiple sclerosis OR cerebral palsy OR cerebral paresis OR poliomyelitis OR polio).ti,kf)

**2) Cardiovascular disease** (myocardial infarction, heart failure, stroke)

(*Cardiovascular Diseases/ OR *Heart Diseases/ OR *Vascular Diseases/ OR *Heart Failure/ OR *"Myocardial Infarction"/ OR exp *Stroke/ OR (((cardiovascular OR cardiac OR heart) ADJ3 (disease* OR disorder* OR event*)) OR cvd OR cvds OR ((heart OR cardia* OR myocard*) ADJ3 (attack* OR fail* OR insufficien* OR infarct*)) OR stroke* OR cardiopath*).ti,kf) OR ((exp *Risk Factors/ OR *Risk/ OR risk*.ti,kf) AND (exp *Cardiovascular Diseases/ OR Heart Failure/ OR Myocardial Infarction/ OR Stroke/))

**3) Risk (factors)**

(exp risk/ OR prevalence/ OR incidence/ OR (risk* OR prevalence* OR incidence* OR predict* OR associat*).ab,ti.)

**4) Study type** (observational studies, cohorts, case-control studies, case-series)

(cohort studies/ OR longitudinal studies/ OR prospective studies/ OR retrospective studies/ OR follow-up studies/ OR case control studies/ OR observational study/ OR (cohort* OR ((prospectiv* OR retrospectiv* OR populat* OR observat* OR (case* ADJ3 control*)) AND (stud*)) OR (case* ADJ3 series) OR ((follow-up* OR followup* OR longitudinal*) AND stud*)).ab,ti.)

**5) Limits** (excluding: conference abstracts, editorials, notes, case reports, (systematic) reviews, meta-analyses; pediatric studies, and animal studies)

**NOT** (news or comment or editorial or congress or review).pt.
**NOT** (systematic review/ or meta-analysis/) **NOT** ((systematic ADJ3 review) OR meta-analys*).ti.) **NOT** (Case reports/ OR case-report*.ti.)

**NOT** ((exp infant/ or exp child/ or adolescent/) not (exp adult/)) **NOT** (child* OR pediatr* OR paediatr* OR infan* OR neonat*).ti.
**NOT** (exp animals/ NOT humans/)

**Web of Science** (Core Collection)
<https://www.webofscience.com/wos/woscc/summary/76015e3b-6bc6-48f7-94d2-531ec7bac0e2-0152c8b642/relevance/1>

Concepts: (1) AND (2) AND (3) AND (4) - NOT (5) (Limits)

**1) Population: People with physical disabilities, including spinal cord injury**

TI=((((physical*) NEAR/3 (disab* OR deficien* OR handicap* OR impair* OR incapac*)) OR ((spine or spinal) NEAR/3 (injur* or trauma* or damag* OR ruptur* OR paralys*)) OR ((brain OR cerebr*) NEAR/3 (injur* OR contus* OR concus* OR damag* OR lesion*)) OR ((individual OR individuals OR patient* OR person* OR people) NEAR/3 (paralys* OR disabilit* OR disabled)) OR ('spinal cord' NEAR/3 (lacerat* OR transect* OR lesion* OR trauma*)) OR (myelopath* NEAR/3 (trauma* OR post-trauma* OR posttrauma*)) OR parapleg* OR quadripleg* OR tetrapleg* OR "spina bifida" OR paralympi* OR para-athlet* OR parathlet* OR para-sport* OR parasport* OR (athlet* NEAR/3 (disabilit* OR disabled)) OR ((wheelchair* OR wheel-chair*) NEAR/3 (user*)) OR (mobilit* NEAR/3 (disabilit*)) OR "musculoskeletal disabilit*" OR ((muscle* OR muscular OR myoton*) NEAR/3 dystroph*) OR myodystroph* OR "multiple sclerosis" OR "cerebral palsy" OR "cerebral paresis" OR poliomyelitis OR polio))

OR

AK=((((physical*) NEAR/3 (disab* OR deficien* OR handicap* OR impair* OR incapac*)) OR ((spine or spinal) NEAR/3 (injur* or trauma* or damag* OR ruptur* OR paralys*)) OR ((brain OR cerebr*) NEAR/3 (injur* OR contus* OR concus* OR damag* OR lesion*)) OR ((individual OR individuals OR patient* OR person* OR people) NEAR/3 (paralys* OR disabilit* OR disabled)) OR ('spinal cord' NEAR/3 (lacerat* OR transect* OR lesion* OR trauma*)) OR (myelopath* NEAR/3 (trauma* OR post-trauma* OR posttrauma*)) OR parapleg* OR quadripleg* OR tetrapleg* OR "spina bifida" OR paralympi* OR para-athlet* OR parathlet* OR para-sport* OR parasport* OR (athlet* NEAR/3 (disabilit* OR disabled)) OR ((wheelchair* OR wheel-chair*) NEAR/3 (user*)) OR (mobilit* NEAR/3 (disabilit*)) OR "musculoskeletal disabilit*" OR ((muscle* OR muscular OR myoton*) NEAR/3 dystroph*) OR myodystroph* OR "multiple sclerosis" OR "cerebral palsy" OR "cerebral paresis" OR poliomyelitis OR polio))

OR

KP=((((physical*) NEAR/3 (disab* OR deficien* OR handicap* OR impair* OR incapac*)) OR ((spine or spinal) NEAR/3 (injur* or trauma* or damag* OR ruptur* OR paralys*)) OR ((brain OR cerebr*) NEAR/3 (injur* OR contus* OR concus* OR damag* OR lesion*)) OR ((individual OR individuals OR patient* OR person* OR people) NEAR/3 (paralys* OR disabilit* OR disabled)) OR ('spinal cord' NEAR/3 (lacerat* OR transect* OR lesion* OR trauma*)) OR (myelopath* NEAR/3 (trauma* OR post-trauma* OR posttrauma*)) OR parapleg* OR quadripleg* OR tetrapleg* OR "spina bifida" OR paralympi* OR para-athlet* OR parathlet* OR para-sport* OR parasport* OR (athlet* NEAR/3 (disabilit* OR disabled)) OR ((wheelchair* OR wheel-chair*) NEAR/3 (user*)) OR (mobilit* NEAR/3 (disabilit*)) OR "musculoskeletal disabilit*" OR ((muscle* OR muscular OR myoton*) NEAR/3 dystroph*) OR myodystroph* OR "multiple sclerosis" OR "cerebral palsy" OR "cerebral paresis" OR poliomyelitis OR polio))

**2) Cardiovascular disease** (myocardial infarction, heart failure, stroke)

TI=((((cardiovascular OR cardiac OR heart) NEAR/3 (disease* OR disorder* OR event*)) OR cvd OR cvds OR ((heart OR cardia* OR myocard*) NEAR/3 (attack* OR fail* OR insufficien* OR infarct*)) OR stroke* OR cardiopath*))

OR

AK=((((cardiovascular OR cardiac OR heart) NEAR/3 (disease* OR disorder* OR event*)) OR cvd OR cvds OR ((heart OR cardia* OR myocard*) NEAR/3 (attack* OR fail* OR insufficien* OR infarct*)) OR stroke* OR cardiopath*))

OR

KP=((((cardiovascular OR cardiac OR heart) NEAR/3 (disease* OR disorder* OR event*)) OR cvd OR cvds OR ((heart OR cardia* OR myocard*) NEAR/3 (attack* OR fail* OR insufficien* OR infarct*)) OR stroke* OR cardiopath*))

**3) Risk (factors)**

TI=((risk* or prevalence* or incidence* or predict* or associat*)) OR AK=((risk* or prevalence* or incidence* or predict* or associat*)) OR KP=((risk* or prevalence* or incidence* or predict* or associat*))

**4) Study type** (observational studies, cohorts, case-control studies, case-series)

TS=((cohort* OR ((prospectiv* OR retrospectiv* OR populat* OR observat* OR (case* NEAR/3 control*)) AND (stud*)) OR (case* NEAR/3 series) OR ((follow-up* OR followup* OR longitudinal*) AND stud*)))

**5) Limits** (excluding: conference abstracts, editorials, notes, case reports, (systematic) reviews, meta-analyses; pediatric studies, and animal studies)

**NOT** TI=((child* OR pediatr* OR paediatr* OR infan* OR newborn* OR neonat*) NOT (adult*))

**NOT** TS=((animal* OR plant* OR rats OR mice OR pigs) NOT (human* OR patient*))

**AND** DT=(article OR early access OR proceeding paper OR letter)

Field Tags: TI=Title, AK=Author Keywords, KP=Keyword Plus®, TS=Topic: searches title, abstract, keyword plus, and author keywords

**Google Scholar** (first 200 results, sorted by relevance)

Searched via: Publish or Perish, Harzing (RRID:SCR_005968)

"cardiovascular|cardiac|heart|myocardial disease|failure|infarction|event|attack"|stroke risk "spinal cord injury"|disability|disabilities|"cerebral palsy"|"multiple sclerosis"|polio|"spina bifida"|amputation cohort|cohorts|nation -cognitive -intellectual
